# Lateral elbow tendinopathy and artificial intelligence: Binary and multilabel findings detection using machine learning algorithms

**DOI:** 10.3389/fmed.2022.945698

**Published:** 2022-09-23

**Authors:** Guillermo Droppelmann, Manuel Tello, Nicolás García, Cristóbal Greene, Carlos Jorquera, Felipe Feijoo

**Affiliations:** ^1^Research Center on Medicine, Exercise, Sport and Health, MEDS Clinic, Santiago, RM, Chile; ^2^Health Sciences Ph.D. Program, Universidad Católica de Murcia UCAM, Murcia, Spain; ^3^Principles and Practice of Clinical Research (PPCR), Harvard T.H. Chan School of Public Health, Boston, MA, United States; ^4^School of Industrial Engineering, Pontificia Universidad Católica de Valparaíso, Valparaíso, Chile; ^5^MSK Diagnostic and Interventional Radiology Department, MEDS Clinic, Santiago, RM, Chile; ^6^Hand and Elbow Unit, Department of Orthopaedic Surgery, MEDS Clinic, Santiago, RM, Chile; ^7^Facultad de Ciencias, Escuela de Nutrición y Dietética, Universidad Mayor, Santiago, RM, Chile

**Keywords:** AUC curve, diagnosis, random forest, tennis elbow, ultrasound

## Abstract

**Background:**

Ultrasound (US) is a valuable technique to detect degenerative findings and intrasubstance tears in lateral elbow tendinopathy (LET). Machine learning methods allow supporting this radiological diagnosis.

**Aim:**

To assess multilabel classification models using machine learning models to detect degenerative findings and intrasubstance tears in US images with LET diagnosis.

**Materials and methods:**

A retrospective study was performed. US images and medical records from patients with LET diagnosis from January 1st, 2017, to December 30th, 2018, were selected. Datasets were built for training and testing models. For image analysis, features extraction, texture characteristics, intensity distribution, pixel-pixel co-occurrence patterns, and scales granularity were implemented. Six different supervised learning models were implemented for binary and multilabel classification. All models were trained to classify four tendon findings (hypoechogenicity, neovascularity, enthesopathy, and intrasubstance tear). Accuracy indicators and their confidence intervals (*CI*) were obtained for all models following a K-fold-repeated-cross-validation method. To measure multilabel prediction, multilabel accuracy, sensitivity, specificity, and receiver operating characteristic (ROC) with 95% *CI* were used.

**Results:**

A total of 30,007 US images (4,324 exams, 2,917 patients) were included in the analysis. The RF model presented the highest mean values in the area under the curve (AUC), sensitivity, and also specificity by each degenerative finding in the binary classification. The AUC and sensitivity showed the best performance in intrasubstance tear with 0.991 [95% *CI*, 099, 0.99], and 0.775 [95% *CI*, 0.77, 0.77], respectively. Instead, specificity showed upper values in hypoechogenicity with 0.821 [95% *CI*, 0.82, −0.82]. In the multilabel classifier, RF also presented the highest performance. The accuracy was 0.772 [95% *CI*, 0.771, 0.773], a great macro of 0.948 [95% *CI*, 0.94, 0.94], and a micro of 0.962 [95% *CI*, 0.96, 0.96] AUC scores were detected. Diagnostic accuracy, sensitivity, and specificity with 95% *CI* were calculated.

**Conclusion:**

Machine learning algorithms based on US images with LET presented high diagnosis accuracy. Mainly the random forest model shows the best performance in binary and multilabel classifiers, particularly for intrasubstance tears.

## Introduction

Lateral elbow tendinopathy (LET) (1), also known as tennis elbow (2), is one of the most frequent musculoskeletal disorders (3). The common extensor tendon, specifically the extensor carpi radialis brevis, is directly involved in the development of this condition (4). LET is a potentially debilitating condition causing significant pain and disability for periods of 12 months or more (5), and in some cases, also generates disruptive sleep (6). This condition is estimated to affect 3.3–3.5 per 1,000 by year (7), affecting individuals during their most productive period (8) and increasing in tennis players with a prevalence of over 40–50% (9). Effective treatment for this tendinopathy is uncertain, with controversial scientific evidence that provides more than 40 modalities (10) in 200 clinical trials and several systematic reviews (11).

Although LET remains primarily a clinical diagnosis (12), the ultrasound (US) findings in common extensor tendon have been well documented in asymptomatic persons (13–17) and LET individuals with tendon structural changes (18–22). However, the degree of these tendon structural changes is highly diverse, with different levels of accuracy (19, 23), making the interpretation of the US imaging a real radiological challenge. For example, a met analysis reported that the US sensitivity and specificity in the detection of common extensor tendon ranged between 64 and 100% and 36 and 100%, respectively (24). Furthermore, this high variability can increase even more if different types of degenerative findings are considered, such as hypoechogenicity, bone changes, neovascularity, calcifications, cortical irregularities (25), and tear (thickness) (26), increasing the lack of precision in the diagnosis by US images. To date, there is still no consensus about what parameters should be considered for the evaluation of changes in the tendon matrix (27).

Recently, artificial intelligence has shown the potential to revolutionize the accuracy of diagnosis by developing a series of classification models (28) and by reducing medical diagnosis variability (29–31). The algorithms based on machine learning and convolutional neural network have been successfully used in pattern recognition in different clinical contexts and specialties, such as neurology (32–34), pulmonary (35–37), cardiovascular (38–42), and oncology (43–51), improving diagnosis accuracy, weighted errors, false-positive rate, sensitivity, specificity, and the area under the receiver operating characteristic curve (AUC) (52). In radiology, machine learning and convolutional neural network algorithms have been used to detect and classify injury patterns in fractures, cartilage defects, meniscal and anterior cruciate ligament tears, and spinal metastases (53, 54) with excellent performance indices.

Most of the studies mentioned above have used computed tomography scan, magnetic resonance imaging, and X-rays as an image-generating source. For example, fracture detection using a computed tomography scan has been used by Tomita et al. (55) with deep neural networks for automatic detection of osteoporotic vertebral fractures, obtaining an accuracy of 89.2%. Another author (56) that also studied automated detection of posterior-element fractures with deep convolutional networks obtained an AUC of 85.7%. There is also some experience using automatic classification and detection of calcaneus fracture with an accuracy of 98% (57). Couteaux et al. (58), Bien et al. (59), and Roblot et al. (60) developed algorithms to automatically detect knee meniscal tears using convolutional neural networks and deep learning assisted with magnetic resonance imaging, obtaining AUC scores of 90.6, 84.7, and 92%, respectively. A similar performance was obtained by authors in (61), where cartilage lesion detection algorithms were developed, reaching accuracy levels of 91%. In radiography, different applications are considered, such as deep learning classification algorithms for the detection of ossification areas of the hand to estimate skeletal maturity (62), obtaining accuracy results similar to an expert radiologist (63). Another publication evaluated knee osteoarthritis in 3,000 subjects (5,960 knees) from the Osteoarthritis Initiative dataset using deep learning techniques. They achieved an AUC of 93%, although the diagnosis is highly dependent on the practitioner’s subjectivity, just like US methods (64). As noted earlier, however, US imaging has not been frequently used as an image-generating source.

Machine learning for the medical US continues to be an opportunity (65), especially in musculoskeletal disorders since the US is highly operator-dependent (66) and the applications are dictated by adequate front-end beamforming, compression, signal extraction, and velocity (67), requiring significant training to acquire a level of competence in clinical diagnosis (68) because the images contain multiplicative noise (69). Baka et al. (70) proposed a model to learn the appearance of the bone interface using US images and random forest methods, obtaining a precision of 86%. Another group proposed an algorithm to segment vertebral US images into three regions with a classification rate of 84.7% (71). In tendon, literature is uncommon yet. In 2017, the University of Salford from the United Kingdom reported in an international conference an automatic method to detect and classify Achilles tendon injuries using decision trees, non-linear support vector machines, and ensemble classifiers (69). Kapinski in 2018 (72) reported a novel method for continuous evaluation of reconstructed Achilles tendon healing based on the responses of intermediate convolutional neural network layers. Note that the task of detecting and classifying different conditions as described above can be considered simple since they are based on binary results (an anomaly can only be present or not) (54). This study differs from others that use deep learning or convolutional neural networks because it uses a multilabel, fast, and simplified classifier to find different degenerative patterns simultaneously, such as hypoechogenicity, neovascularity, bony irregularities, and fibrillar disruptions. Currently, no scientific publications have identified ultrasonographic findings using artificial intelligence algorithms.

This article aims to assess multilabel classification models using machine learning algorithms to detect degenerative findings and intrasubstance tear in US images with LET diagnosis.

## Materials and methods

### Study design

This study was designed as a retrospective and multicentric study. It was written following the Strengthening the Reporting of Observation studies in Epidemiology (STROBE) guideline (73). All patients records with an elbow US exam at MEDS Clinic in Santiago, Región Metropolitana, Chile. This study started on March 1st, 2019.

### Subjects

**FIGURE 1:**
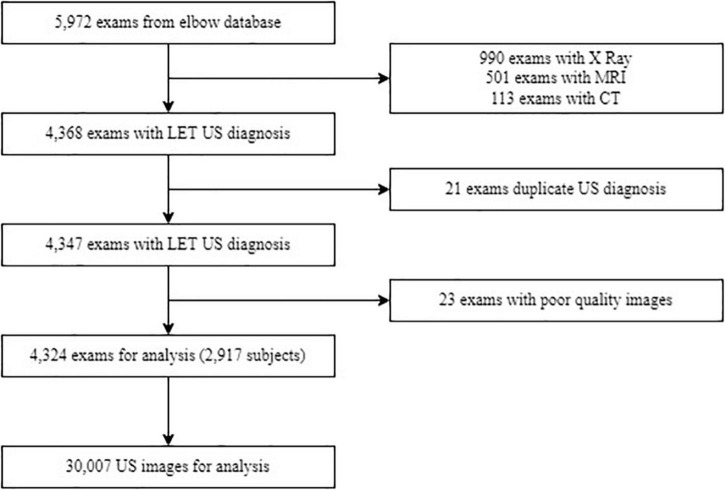
Flowchart of data selection and subjects used in the study. Abbreviations: MRI, magnetic resonance imaging; CT, computed tomography scan; LET, lateral elbow tendinopathy; US, ultrasound.

Only images of the common extensor tendon were considered. We selected US images and medical records from patients with a LET diagnosis from January 1st, 2017, to December 30th, 2018. The inclusion criteria were: (1) clinical diagnosis of LET established by orthopedists, sports medicine physicians, or any musculoskeletal specialists, (2) US exam made in the medical center of interest, (3) US exam reported by any musculoskeletal radiologist with more than 10 years of experience, and (4) no race or age restriction. Consecutively, exclusion criteria were: (1) US-guided procedures, such as corticoid, stem cell, and platelet-rich plasma injections, (2) previous LET surgery, and (3) duplicate or not distinguishable images, were removed from the dataset. Figure 1 provides the flowchart to select the subjects.

### Ultrasound assessment of common extensor tendon

All common extensor tendons were assessed using an Aplio 500 US system (Toshiba America Medical Systems, Inc, Tustin, CA, USA) equipped with a multifrequency linear transducer was used. A frequency of 18 MHz was chosen. The images were stored as Digital Imaging and Communications in Medicine (DICOM) files and reviewed on a picture archiving and communication system (PACS).

All patients with LET diagnosis were examined in a seated position with flexion elbow in 90 grades with the wrist pronated, and the arm was resting on a table (14).

**FIGURE 2:**
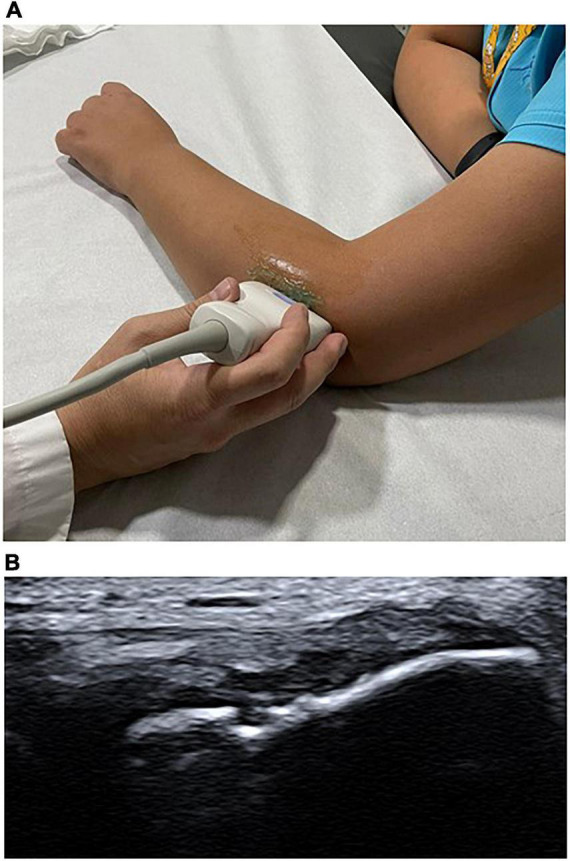
Patient evaluation position and an ultrasound (US) finding, respectively. **(A)** Probe positioning in the elbow in the US exploration of the extensor tendon complex. **(B)** US imaging shows intrasubstance tear in extensor tendon complex.

Greyscale and color Doppler US imaging are standard methods used for assessing tendon structural changes (74). Following the literature recommendations, four common prevalent degenerative findings were selected from US exams, such as hypoechogenicity, neovascularity, enthesopathy, and intrasubstance tear (75). A focal hypoechoic region was defined as being rounded and not associated with tendon disruption. Neovascularity was assessed as the presence of blood flow on color Doppler. Enthesopathy was evaluated as bony abnormalities at the tendon insertion. A linear intrasubstance tear was defined as a linear hypoechoic focus associated with discontinuity of tendon fibers (76–80). Every finding was evaluated with a binary score as present or absent. We recorded when an exam presents more than one degenerative finding. Figure 2A shows the evaluation position, and Figure 2B represents US finding, in this case, an intrasubstance tear.

### Datasets: Ultrasound image and database

**FIGURE 3:**
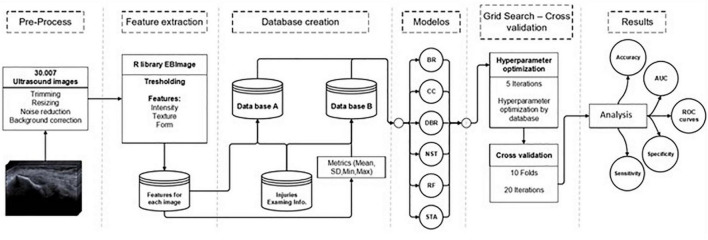
Study workflow. Abbreviations: BR, binary relevance model; CC, classifier chains model; DBR, dependent binary relevance model; NST, nested stacking model; RF, random forest; STA, staking generalization; AUC, area under the curve.

Several recommendations were followed for data (images) pre-processing, object detection, and feature extraction (81–83). Two datasets (A and B) were built for training and testing models. The pre-processing step considers eliminating any elements that generated noise in the images, such as uneven lighting, different sizes, or image portions without information (84). Object detection is a specific injury area of interest for the analysis. However, in this case, we considered the common extensor tendon image. Feature extraction is an important step in the construction of any pattern classification and aims at the extraction of the relevant information that characterizes each class (85). According to the 7th International Conference on System Engineering and Technology 2017, texture analysis and classification in US medical images can use feature extraction and texture characteristics for determining echo pattern characteristics (86). One of the most used are intensities distribution (mean intensity and standard deviation), pixel-pixel co-occurrence patterns, and scales granularity. Then the shape contour was extracted where the texture of the pixels was quantified. The US images were labeled manually with four degenerative findings classification outputs findings (hypoechogenicity, neovascularity, enthesopathy, and intrasubstance tear) (65) and complementary patient data such as sex, age, and side of the injury (right or left). The final process consists of a combination between the patient’s information and image analysis. Dataset A was image prediction and contained data extraction from 95 morphology characteristics, shapes, and texture variables, where one image corresponding to one diagnostic (30.007 rows). Dataset B was the patient prediction and included 380 variables from data extraction, such as median, standard deviation, minimal, and maximal, where one exam corresponds to one diagnostic (4.321 rows). Figure 3 represents the study workflow process.

### Machine learning and statistical analysis

Supervised learning was used because most machine learning applications for US involve them. Both datasets were implemented into binary and multilabel classification algorithms in six machine learning methods: Binary relevance model, classifier chains model, nested stacking model, dependent binary relevance model, staking generalization, and random forest.

All models were trained to classify four tendon findings (hypoechogenicity, neovascularity, enthesopathy, and intrasubstance tear) in images with LET diagnosis. First, each pattern was recognized individually and then the four finding simultaneously. Different metrics were conducted to assess the classification of machine learning models. A K-fold-repeated-cross-validation (KFRCV) with ten as the number of folds was used. After this process, means and confidence intervals (*CI*) values were obtained.

Data were analyzed using R version 3.6.2 (R Foundation for Statistical Computing). The following packages were used: “EBImage” for characteristics extraction, “mlr” for each machine learning algorithm, and “randomForest” for the random forest (87–89). Additionally, to measure multilabel prediction (classification) were used multilabel accuracy, sensitivity, specificity, and receiver operating characteristic (ROC) (90). Also, we included a positive predictive value. Differences in US findings between women and men were assessed for significance using the *T*-test and chi-squared test. The significance level was considered *p* < (0.05) and 95% *CI* for all metrics.

## Results

### Common extensor tendinopathy

A total of 30,007 US images, 6.9 on average in 4,324 exams, and medical records from 2,917 patients with a LET diagnosis were included in the data analysis in this study. Patients’ age was presented with a minimum value of 7 and a maximum of 91 years. Women are older than men in 1 year 47.18 ± 11.00 (*p* < 0.001) and also, they presented statistical differences in hypoechogenicity finding in comparison with men (*p* = 0.01). The total of exams presented at least one degenerative finding. US features are summarized in Table 1.

**TABLE 1:** Ultrasound findings comparison between sexes.

Demographic characteristics/ degenerative findings	Female (*N* = 1717) Mean ± SD; *n* (%)	Male (*N* = 2607) Mean ± SD; *n* (%)	*p*-value	Total (*N* = 4324) Mean ± SD; *n* (%)
Age	47.18 ± 11.00	45.99 ± 11.03	<0.001^a^	46.46 ± 11.03
Right side of the injury	1179 (68.88)	1790 (68.66)	0.98	2969 (68.66)
HE	1201 (69.94)	1730 (66.35)	0.0119^b^	2931 (67.75)
NV	636 (37.04)	999 (38.31)	0.4093	1635 (37.79)
E	599 (34.88)	915 (35.09)	0.9411	1514 (35.00)
IST	582 (33.89)	880 (33.75)	0.9521	1462 (33.80)

HE, hypoechogenicity; NV, neovascularity; E, enthesopathy; IST, intrasubstance tear. ^*a*^*p*-value < 0.001. ^*b*^*p*-value < 0.01.

### Machine learning models for a binary classifier

Table 2 shows the binary classification performance (AUC, sensitivity, and specificity) for both datasets (A and B) in each of the six machine learning algorithms. Main degenerative findings in LET (hypoechogenicity, neovascularity, enthesopathy, and intrasubstance tear) were considered under analysis. Focusing on AUC sensitivity and specificity, most models performed with variability among them. Results were described in most cases with a minimal range of 95% *CI*, demonstrating a robust performance for all models. Notably, the RF model obtained the best results. For example, Table 2 shows dataset A, where random forest presented the highest mean values in AUC, sensitivity, and also specificity by each degenerative finding. The AUC and sensitivity showed the best performance in IST with 0.991 [95% *CI*, 0.99, −0.99], and 0.775 [95% *CI*, 0.77, −0.77], respectively. Instead, specificity showed upper values in hypoechogenicity with 0.821 [95% *CI*, 0.82, −0.82].

**TABLE 2:** The area under the curve (AUC), sensitivity, and specificity [95% *CI*] values of six machine learning classifiers based on degenerative findings in datasets A and B.

Dataset	Measure	Model	HE [95% *CI*]		NV [95% *CI*]		E [95% *CI*]		IST [95% *CI*]	
A	AUC	BR	0.806	(0.81, 0.81)	0.901	(0.900, 0.902)	0.7482	(0.747, 0.749)	0.963	(0.963, 0.964)
		CC	0.810	(0.81, 0.81)	0.897	(0.896, 0.898)	0.6954	(0.689, 0.701)	0.961	(0.960, 0.963)
		DBR	0.804	(0.8, 0.81)	0.892	(0.891, 0.893)	0.6488	(0.647, 0.650)	0.956	(0.954, 0.958)
		NST	0.806	(0.81, 0.81)	0.901	(0.900, 0.902)	0.7463	(0.745, 0.747)	0.963	(0.963, 0.964)
		RF	0.928	(0.93, 0.93)	0.974	(0.973, 0.974)	0.8993	(0.898, 0.9)	0.991	(0.990, 0.991)
		STA	0.806	(0.81, 0.81)	0.847	(0.846, 0.848)	0.688	(0.686, 0.689)	0.935	(0.934, 0.936)
	SE	BR	0.577	(0.58, 0.58)	0.704	(0.703, 0.704)	0.6568	(0.656, 0.657)	0.760	(0.759, 0.760)
		CC	0.578	(0.58, 0.58)	0.702	(0.701, 0.703)	0.6234	(0.619, 0.627)	0.759	(0.758, 0.76)
		DBR	0.576	(0.58, 0.58)	0.699	(0.698, 0.7)	0.594	(0.593, 0.595)	0.756	(0.754, 0.757)
		NST	0.577	(0.58, 0.58)	0.704	(0.703, 0.704)	0.6556	(0.654, 0.656)	0.760	(0.759, 0.760)
		RF	0.607	(0.61, 0.61)	0.741	(0.740, 0.741)	0.7522	(0.751, 0.752)	0.775	(0.774, 0.776)
		STA	0.577	(0.58, 0.58)	0.676	(0.676, 0.677)	0.6187	(0.617, 0.619)	0.744	(0.743, 0.744)
	SP	BR	0.729	(0.73, 0.73)	0.697	(0.696, 0.697)	0.5913	(0.590, 0.591)	0.703	(0.702, 0.70)
		CC	0.732	(0.73, 0.73)	0.695	(0.694, 0.696)	0.5719	(0.569, 0.574)	0.702	(0.701, 0.703)
		DBR	0.728	(0.73, 0.73)	0.692	(0.691, 0.693)	0.5548	(0.554, 0.555)	0.700	(0.699, 0.701)
		NST	0.729	(0.73, 0.73)	0.697	(0.696, 0.697)	0.5906	(0.590, 0.591)	0.703	(0.702, 0.704)
		RF	0.820	(0.82, 0.82)	0.732	(0.732, 0.733)	0.6469	(0.646, 0.647)	0.715	(0.714, 0.716)
		STA	0.729	(0.73, 0.73)	0.670	(0.670, 0.671)	0.5692	(0.568, 0.569)	0.691	(0.690, 0.691)
B	AUC	BR	0.830	(0.83, 0.83)	0.925	(0.923, 0.927)	0.7811	(0.778, 0.784)	0.960	(0.957, 0.963)
		CC	0.830	(0.83, 0.83)	0.906	(0.901, 0.911)	0.7228	(0.714, 0.731)	0.964	(0.961, 0.966)
		DBR	0.788	(0.79, 0.79)	0.846	(0.842, 0.85)	0.6477	(0.643, 0.652)	0.965	(0.963, 0.967)
		NST	0.830	(0.83, 0.83)	0.926	(0.925, 0.928)	0.781	(0.777, 0.784)	0.960	(0.957, 0.963)
		RF	0.888	(0.89, 0.89)	0.965	(0.964, 0.966)	0.8517	(0.849, 0.854)	0.986	(0.985, 0.987)
		STA	0.829	(0.83, 0.83)	0.870	(0.866, 0.873)	0.7222	(0.717, 0.726)	0.937	(0.935, 0.940)
	SE	BR	0.606	(0.61, 0.61)	0.764	(0.762, 0.765)	0.6821	(0.679, 0.684)	0.804	(0.801, 0.806)
		CC	0.606	(0.61, 0.61)	0.752	(0.749, 0.755)	0.6444	(0.638, 0.650)	0.807	(0.804, 0.809)
		DBR	0.592	(0.59, 0.59)	0.714	(0.712, 0.717)	0.5957	(0.592, 0.598)	0.807	(0.805, 0.809)
		NST	0.606	(0.61, 0.61)	0.765	(0.763, 0.766)	0.6821	(0.679, 0.684)	0.804	(0.801, 0.806)
		RF	0.624	(0.62, 0.63)	0.789	(0.787, 0.790)	0.7279	(0.725, 0.73)	0.821	(0.820, 0.823)
		STA	0.605	(0.6, 0.61)	0.729	(0.727, 0.732)	0.6441	(0.640, 0.647)	0.789	(0.787, 0.791)
	SP	BR	0.723	(0.72, 0.73)	0.660	(0.658, 0.661)	0.5983	(0.597, 0.599)	0.654	(0.653, 0.656)
		CC	0.723	(0.72, 0.73)	0.653	(0.651, 0.655)	0.5779	(0.574, 0.580)	0.656	(0.654, 0.657)
		DBR	0.695	(0.69, 0.7)	0.630	(0.628, 0.632)	0.5516	(0.550, 0.553)	0.656	(0.655, 0.658)
		NST	0.723	(0.72, 0.73)	0.660	(0.659, 0.662)	0.5983	(0.597, 0.599)	0.654	(0.653, 0.656)
		RF	0.763	(0.76, 0.76)	0.675	(0.673, 0.676)	0.623	(0.621, 0.624)	0.663	(0.662, 0.665)
		STA	0.723	(0.72, 0.73)	0.639	(0.637, 0.641)	0.5776	(0.576, 0.579)	0.647	(0.645, 0.648)

AUC, area under the curve; SE, sensitivity; SP, specificity; HE, hypoechogenicity; NV, neovascularity; IST, intrasubstance tear; E, enthesopathy; BR, binary relevance model; CC, classifier chains model; NST, nested stacking model; DBR, dependent binary relevance model; STA, staking generalization; RF, random forest.

A similar situation occurred for dataset B, which showed slightly lower values for the same findings and models. The RF model also demonstrated the best performance for all measures and degenerative features. Table 2 showed the highest AUC and sensitivity values for ISR 0.937 [95% *CI*, 0.93–0.94] and 0.82 [95% *CI*, 0.82, −0.82]. Hypoechogenicity also presented better specificity than other degenerative findings with 0.763 [95% *CI*, 0.72, −0.72].

### Machine learning models for a multilabel classifier

In the previous results section, the machine learning models assessed a binary classification for each degenerative finding. Now, these methods used a multilabel classifier to identify the four types of tendon findings simultaneously in both datasets. In this scenario, the diagnosis presented different accuracy levels in all machine learning models. When the diagnosis was based on the combination of degenerative findings, the random forest algorithm again presented the best performances among the selected models. Table 3 shows that the random forest in dataset A presented the highest multilabel accuracy value of 0.772 [95% *CI*, 0.771, 0.773]. Similarly, in the condition represented in dataset B, these results show that the model performs well in testing environments without presenting overfitting issues. Multilabel accuracy value was 0.723 [95% *CI*, 0.721, 0.726]. Additionally, high macro and micro-AUC scores are observed in RF models in both datasets. These results could be explained due to the balance between sensitivity and specificity shown in RF models. Particularly, micro-AUC observed in dataset A of 0.962 [95% *CI*, 0.962–0.963] and 0.942 [95% *CI*, 0.941–0.943] in dataset B results are essential because aggregating the contributions of all classes to compute the average metric.

**TABLE 3:** Multilabel accuracy values of six machine learning classifiers based on degenerative findings in both datasets.

Dataset	Model	Macro AUC		Micro AUC		SE		SP		Accuracy		PPV	
A	BR	0.854	(0.854, 0.855)	0.911	(0.910, 0.911)	0.700	(0.700, 0.701)	0.710	(0.71, 0.710)	0.683	(0.682, 0.684)	0.818	(0.816, 0.821)
	CC	0.841	(0.839, 0.842)	0.891	(0.889, 0.893)	0.691	(0.690, 0.692)	0.700	(0.699, 0.701)	0.691	(0.689, 0.692)	0.790	(0.783, 0.798)
	DBR	0.825	(0.824, 0.826)	0.865	(0.864, 0.866)	0.678	(0.678, 0.678)	0.687	(0.686, 0.687)	0.697	(0.696, 0.698)	0.765	(0.7630, 766)
	NST	0.854	(0.853, 0.854)	0.910	(0.910, 0.911)	0.700	(0.700, 0.700)	0.710	(0.709, 0.710)	0.683	(0.682, 0.684)	0.818	(0.816, 0.821)
	RF	0.948	(0.947, 0.948)	0.962	(0.962, 0.963)	0.725	(0.725, 0.726)	0.736	(0.736, 0.737)	0.772	(0.771, 0.773)	0.891	(0.890, 0.892)
	STA	0.819	(0.818, 0.819)	0.897	(0.897, 0.898)	0.694	(0.693, 0.694)	0.703	(0.703, 0.703)	0.683	(0.682, 0.684)	0.818	(0.816, 0.821)
B	BR	0.874	(0.872, 0.875)	0.918	(0.917, 0.919)	0.735	(0.734, 0.736)	0.682	(0.681, 0.682)	0.665	(0.662, 0.668)	0.804	(0.799, 0.809)
	CC	0.855	(0.853, 0.85)	0.899	(0.897, 0.902)	0.725	(0.724, 0.726)	0.674	(0.673, 0.675)	0.676	(0.673, 0.679)	0.777	(0.772, 0.783)
	DBR	0.811	(0.809, 0.813)	0.847	(0.845, 0.849)	0.696	(0.694, 0.697)	0.651	(0.650, 0.652)	0.658	(0.656, 0.661)	0.770	(0.765, 0.775)
	NST	0.874	(0.873, 0.876)	0.918	(0.917, 0.919)	0.736	(0.735, 0.737)	0.682	(0.681, 0.683)	0.666	(0.663, 0.669)	0.804	(0.799, 0.810)
	RF	0.922	(0.921, 0.923)	0.942	(0.941, 0.943)	0.749	(0.748, 0.750)	0.692	(0.692, 0.693)	0.723	(0.721, 0.726)	0.858	(0.855, 0.862)
	STA	0.839	(0.838, 0.841)	0.898	(0.897, 0.899)	0.724	(0.723, 0.725)	0.673	(0.672, 0.674)	0.663	(0.660, 0.665)	0.808	(0.802, 0.814)

AUC, area under the curve; SE, sensitivity; SP, specificity; PPV, positive predictive value; BR, binary relevance model; CC, classifier chains model; NST, nested stacking model; DBR, dependent binary relevance model; STA, staking generalization; RF, random forest.

### Diagnosis performance

**FIGURE 4:**
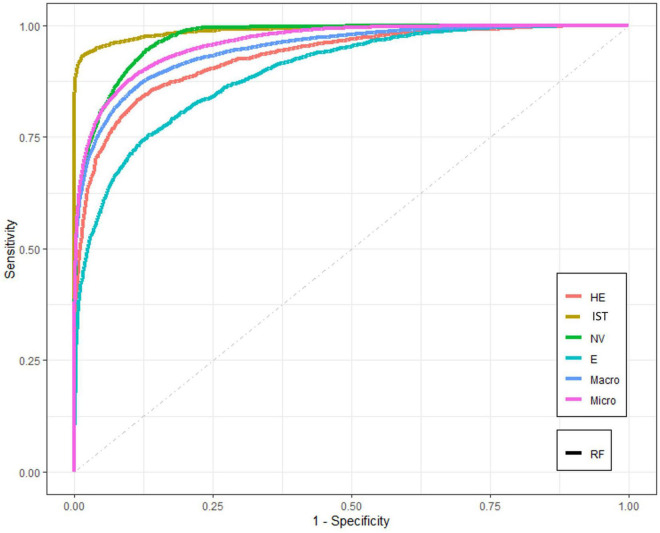
The receiver operating characteristic (ROC) curves for RF model for dataset A. Abbreviations: RF, random forest; HE, hypoechogenicity; NV, neovascularity; IST, intrasubstance tear; E, enthesopathy; Macro, macro-AUC; Micro, micro-AUC.

Figure 4 represents dataset A, and the results show the relation between sensitivity vs. 1-specificity across each degenerative finding using the random forest model. In this figure, the plot shows the higher discriminant capacity of diagnosis detection. Most of the lines are located progressively closer to the upper left-hand corner in ROC space. The intrasubstance tear shows the most significant discriminate capacity in comparison with the other tendon injuries. However, the enthesopathy finding presented the lowest discriminate capacity in this model.

## Discussion

This study is one of the first to present multilabel classification models using machine learning algorithms to detect degenerative findings and intrasubstance tear in US images with LET diagnosis. This retrospective analysis explicitly considered one of the most extensive series of extensor carpi radialis brevis US images, and our machine learning-based tool for diagnosis of LET was trained using the largest dataset so far. The most notable outcomes in this study were obtained by incorporating several machine learning models based on diagnosis know condition. Excellent results and highest values for all degenerative findings were detected in the binary classification performance. Moreover, when the US diagnosis was based on the combination of degenerative findings using a multilabel classifier, the accuracy values presented strong performance too. Our results showed that the random forest algorithm presented the best diagnosis performance, in both binary and multilabel models. These results demonstrate that the implementation of tools derived from artificial intelligence can be used to support the imaging for tendinopathies. Collaborative work between the radiologist and the algorithm could improve the precision of the results, especially if the institution does not have a radiologist specializing in the musculoskeletal area.

Traditionally, US has been demonstrated as a cost-effective tool for detecting abnormalities patterns in tendon structures. Additionally, there is evidence to support the use of US in the detection of LET. A meta-analysis published in 2014 determined that diagnostic test accuracy appears to be highly dependent on numerous variables, such as operator experience, equipment, and stage of pathology. However, US has variable sensitivity and specificity (sensitivity: 64–100%; specificity: 36–100%), decreasing the clinical diagnosis precision (24). Another article published in the same year reported specifically the sensitivity and specificity for each abnormal US finding using traditional detection method. The hypoechogenicity presented the best combination of diagnostic sensitivity and specificity. It is moderately sensitive sensitivity: 0.64 [95% *CI*, 0.56, 0.72] and highly specific specificity 0.82 [95% *CI*, 0.72, 0.90]. Additionally, neovascularity specificity 1.00 [95% *CI*, 0.97, 1.00)], calcifications specificity 0.97 [95% *CI*, 0.94, 0.99], and cortical irregularities specificity 0.96 [95% *CI*, 0.88, 0.99] have strong specificity for chronic lateral epicondylalgia (25). Our results, particularly for intrasubstance tear detection using the binary algorithm classification in both datasets, demonstrated a superior performance to the traditional US diagnosis methods. In the case of multilabel accuracy, the performance for both indicators was lowest results of specificity and sensitivity than the binary method. This situation could be explained because it is difficult to find a function that minimized the error for more classes. In other words, it increases the variability of the response variable.

For example, in the binary classification, the enthesopathy presented the lowest performance of the six machine learning classifiers. Notably, in the dependent binary relevance model from dataset B, our analysis showed that AUC was 0.647 [95% *CI*, 0.64, 0.65]. This result is quite similar to other reports with a sensitivity of 0.65 and specificity of 0.86 for this finding (77). However, our best result in the binary classification was detecting intrasubstance tear injuries using random forest algorithms. The performance showed an AUC of almost 1.0 (0.99) [95% *CI*, 0.99, 0.99] in contrast with the traditional US methods diagnosis for detecting common extensor tendon tear in the lateral with lower performances in sensitivity, specificity, and accuracy with 64.52, 85.19, and 72.73%, respectively (26).

However, one of our research strengths is the execution of machine learning models using multilabel detection for tendon injury findings. To date, few experiences had been published in the musculoskeletal area using artificial intelligence for tendon pattern detection. Some previous experiences have used Automatic ROI Detection and Classification of the Achilles Tendon ultrasound Images (69), and deep learning models for automatic tracking of the muscle-tendon junction or even measuring muscle atrophy (91). Other disciplines have also used other classification techniques such as neural networks or deep learning convolutional neural networks for image detection, demonstrating excellent results. However, CNN and DL have some drawbacks that should be analyzed when developing predictive models. First, it has been shown that DL requires large datasets to obtain better performance. To handle this, transfer learning is commonly used. However, DL architectures should also be re-trained and model parameters should be optimized, looking out for possible overfitting patterns. Second, DL architectures rely on the high computational performance, and it takes longer to prove results. In this sense, they are more complex to implement, especially in a clinical environment with a high demand for care, so improving diagnostic speed without compromising diagnostic accuracy is crucial for patients and the health system. Therefore, machine learning algorithms are advantageous when speed is of interest. In this case, the execution times of the proposed method were very low, allowing it to be easily implemented in a hospital scenario and re-trained with new data that is daily generated. Finally, the multilabel classification model differs from other algorithms most commonly used in image diagnosis due to the simplicity of its implementation.

This study also has some limitations. Firstly, our images come from the same institution, and patients presented similar socioeconomic conditions. Secondly, we included all static US images from common extensor tendon US per patient, not considering real-time and other structures or tissues. Thirdly, we included tendons with a definitive LET diagnosis, and we did not compare inter and intraobserver variability between radiologists. Fourthly, we considered all images without a region of interest, such as most of the publications. Nevertheless, in a short time, it could be a potential advantage. Finally, we did not repeat the US diagnosis to reduce retrospective bias. However, our radiologist presented more than 10 years of experience.

In conclusion, the random forest model presented the highest sensitivity and specificity in binary and multilabel classifiers for degenerative findings in the common extensor tendon. In particular, intrasubstance tear detections obtained the best performance. Machine learning models could be used to support the US diagnosis of LET.

## Data availability statement

The original contributions presented in this study are included in the article/supplementary material, further inquiries can be directed to the corresponding author.

## Ethics statement

This study has been performed following the latest version of the Declaration of Helsinki and the Chilean scientific legislation. The study was approved by the “Comité de Ética Científico Adulto del Servicio Metropolitano Oriente de la ciudad de Santiago de Chile (SSMO).” The Ethics Committee required no informed consent given the nature of the study. The project was approved on August 7th, 2018. No approval number was recorded.

## Author contributions

GD: conceptualization, data curation, formal analysis, investigation, methodology, validation, and writing the original draft. MT: data curation, formal analysis, investigation, and visualization. NG: validation, review, and editing. CG: review and editing. CJ: resources and validation. FF: conceptualization, formal analysis, investigation, supervision, review, and editing. All authors contributed to the article and approved the submitted version.
